# Coronary Flow Reserve in Adults: Pathophysiology, Assessment Modalities, Clinical Applications, and Prognostic Significance

**DOI:** 10.3390/medicina62061035

**Published:** 2026-05-26

**Authors:** Konstantinos Katogiannis, Jimmy T. Efird, Artur Dziewierz, Francisco Epelde, Ignatios Ikonomidis

**Affiliations:** 1 2nd Cardiology Department, General Hospital “Attikon”, National and Kapodistrian University of Athens, 12462 Athens, Greece; 2 VA Cooperative Studies Program Coordinating Center, Massachusetts Veterans Epidemiology Research and Information Collaborative (MAVERIC), Boston, MA 02111, USA; jimmy.efird@va.gov; 3 School of Medicine, Case-Western Reserve University, Cleveland, OH 44206, USA; 4 Clinical Department of Cardiology and Cardiovascular Interventions, University Hospital, 30-688 Krakow, Poland; adziewierz@gmail.com; 5 Second Department of Cardiology, Institute of Cardiology, Jagiellonian University Medical College, 30-688 Krakow, Poland; 6 Internal Medicine Department, Parc Taulí Hospital Universitari, Institut d’Investigació i Innovació Parc Taulí (I3PT-CERCA), Universitat Autònoma de Barcelona, 08208 Sabadell, Spain; fepelde@gmail.com

**Keywords:** coronary flow reserve, coronary microvascular dysfunction, ischemia with no obstructive coronary arteries (INOCA), fractional flow reserve, prognosis, cardiovascular imaging

## Abstract

Coronary flow reserve (CFR) is a fundamental physiological index defined as the ratio of maximal coronary blood flow during hyperemia to resting flow. It provides an integrated assessment of the entire coronary circulation, from epicardial arteries to the microvasculature. Non-invasive assessment, particularly with transthoracic Doppler echocardiography (TTDE), is valuable in clinical practice for evaluating the functional impact of moderate obstructive lesions and determining the status of coronary microcirculation. Impairment of coronary microcirculation, detected by reduced CFR, is present in diverse conditions such as Tako-Tsubo cardiomyopathy, cardiac syndrome X, hypertension, and slow coronary flow. CFR also serves as a non-invasive tool to examine the effects of various interventions. CFR can be assessed invasively using Doppler guidewire or thermodilution techniques and non-invasively using transthoracic Doppler echocardiography, PET, CMR, CT perfusion, and dynamic SPECT. Lower CFR is observed with advancing age, in females, and in individuals of African descent. An impaired CFR is a powerful, independent predictor of major adverse cardiovascular events (MACEs) across a wide spectrum of diseases, including stable obstructive coronary artery disease (CAD), ischemic syndromes with no obstructive coronary arteries (INOCAs), heart failure, cardiomyopathies, and systemic diseases like diabetes and chronic kidney disease. Beyond risk stratification, CFR is used to guide therapeutic decisions, including revascularization strategies and tailoring of pharmacological interventions. The integration of CFR assessment into clinical practice, supported by recent guideline recommendations, represents a shift towards personalized, physiology-based cardiovascular care.

## 1. Introduction

The evaluation of coronary artery disease (CAD) has undergone a profound transformation over recent decades, shifting decisively from purely anatomical assessment to physiological principles [1,2]. Historically, coronary angiography served as the diagnostic cornerstone. However, evidence reveals significant discordance between a coronary lesion’s anatomical appearance and its hemodynamic impact [3]. This recognition has catalyzed a revolution, redirecting focus from lesion appearance to functional significance.

Central to this transition toward physiology-guided assessment is coronary flow reserve (CFR). Unlike pressure-based indices that primarily assess epicardial conduits, CFR provides an integrated evaluation of the entire coronary vascular tree—from large epicardial arteries to the intricate microvascular network [4,5,6]. This comprehensive assessment is critical, as many patients present with ischemic symptoms despite angiographically normal epicardial coronaries, a condition termed ischemia with non-obstructive coronary arteries (INOCA), frequently resulting from coronary microvascular dysfunction (CMD) [6,7,8,9,10].

Non-invasive assessment of CFR has recently become feasible with Doppler echocardiography [5,11]. To evaluate coronary flow in the left anterior descending (LAD) artery, blood flow at the distal segment is detected by color Doppler, which is feasible in >90% of subjects thanks to advanced technology [11]. CFR is defined as the ratio of maximum diastolic flow velocity (after vasodilation) to diastolic flow velocity at baseline [4]. A CFR of LAD < 2 is indicative of hemodynamically significant stenosis or microcirculatory impairment [12,13].

CFR has evolved from a foundational concept to an important clinical tool, with major guidelines now endorsing comprehensive physiological assessment, particularly in patients with INOCA, suspected coronary microvascular dysfunction, and intermediate coronary lesions [3]. This review examines the pathophysiology, demographic epidemiology, invasive and non-invasive assessment modalities, and the robust prognostic significance of CFR in adult cardiovascular medicine.

## 2. Demographics and Epidemiology of Reduced Coronary Flow Reserve

### 2.1. Introduction

Reduced coronary flow reserve (rCFR) is unevenly distributed across age, sex, and ethnicity [14]. Understanding its epidemiology aids prevention and therapy, especially when microvascular ischemia is present [15].

### 2.2. Epidemiologic Risk Factors and Comorbid Conditions

rCFR indicators include advanced age, female sex, and African descent [16,17,18], often with diabetes, hypertension, obesity, heart failure, inflammation, or smoking [18]. rCFR may precede obstructive CAD—supporting early detection, particularly in diabetes or HFpEF [19,20,21,22]. Systemic inflammation is increasingly linked to coronary microvascular dysfunction [23].

#### 2.2.1. Age-Related Decline in CFR

rCFR rises with age due to reduced vasodilation, arterial stiffness, microvessel rarefaction, and higher oxygen demand [23,24]. CFR peaks at ages 20–30, declines notably from 46 to 60 years, and often becomes abnormal after 75, even without CAD [25,26,27,28].

#### 2.2.2. Sex Differences

Women face higher rCFR risk independent of obstructive CAD [28,29,30,31,32,33]. Nearly half of women have low CFR vs. <30% of the general population [33]. Women more often have chest pain without CAD and are prone to CMD [33,34,35]. Estrogen plays a protective vasodilatory role [36]; postmenopausal women show more rCFR [37,38]. However, rCFR-associated mortality risk does not differ by sex [39].

#### 2.2.3. Ethnic and Racial Variability

Individuals of African descent are disproportionately affected due to hypertension, LV hypertrophy, endothelial dysfunction, and arterial stiffness [40,41]. Other high-risk groups include South Asian (insulin resistance/central obesity) and Native American (diabetes/metabolic syndrome) people [42,43]. East Asian people often have better-preserved CFR [44]. Genetic and social factors contribute [45,46,47,48].

### 2.3. Epidemiologic Limitations

rCFR epidemiology remains poorly defined, with subjective or observational data. Studies often overlook biases, collider effects, residual confounding, small samples, and non-probabilistic sampling [49,50,51,52].

## 3. Pathophysiology and Assessment Modalities of CFR

### 3.1. The Physiological Basis of Coronary Flow Reserve (Figure 1)

CFR is formally defined as the ratio of maximal coronary blood flow during pharmacologically induced hyperemia to basal flow [53]. It represents the functional capacity of the coronary circulation to increase blood supply to meet augmented myocardial oxygen demand. Normally, flow can increase 3–5 times above baseline [54,55,56]. CFR is an integrated assessment determined by cumulative resistance across the entire vascular pathway, encompassing both epicardial arteries and microcirculation [57]. While this holism is a strength, it is also a limitation, as an abnormal CFR cannot distinguish between epicardial and microvascular impairment [53,56,57,58]. However, CFR values between 2 and 2.5 usually suggest coronary microcirculatory disease with absence of a coronary obstruction greater than 70%.

Throughout this manuscript, the general term ‘coronary flow reserve (CFR)’ refers to the ratio of maximal to resting coronary blood flow, whether measured as absolute flow (mL/min or mL/g/min) or as flow velocity. When specifically referring to Doppler-derived velocity measurements, we use the term ‘coronary flow velocity reserve (CFVR)’. PET, CMR, and thermodilution techniques provide estimates of absolute flow or relative flow reserve, while transthoracic Doppler echocardiography provides CFVR. Unless otherwise specified, ‘CFR’ is used generically to encompass all modalities, with modality-specific terms (CFVR, CFRthermo, MFR, MPR) introduced where distinction is necessary.

It is important to note that the numerical threshold defining ‘impaired’ CFR varies by assessment modality and clinical context. For invasive Doppler guidewire measurements, CFVR < 2.0 is generally considered abnormal. For invasive thermodilution, CFRthermo < 2.0 or <2.5 is used depending on the specific protocol and reference standards. For non-invasive PET, a global CFR (or MFR) < 2.0 is typically considered abnormal, although some studies use < 1.8 or <2.5 depending on the tracer and analysis method. For transthoracic Doppler echocardiography, CFVR < 2.0 indicates hemodynamically significant stenosis or microvascular impairment, while values between 2.0 and 2.5 are considered borderline or suggestive of microvascular disease in the absence of significant epicardial obstruction. These variations arise from differences in measurement principles (absolute flow vs. velocity), the specific coronary territory assessed, the hyperemic stimulus, and patient populations. In clinical practice, each laboratory should use modality-specific, validated cutoffs, and serial assessments should employ the same technique.

**Figure 1:**
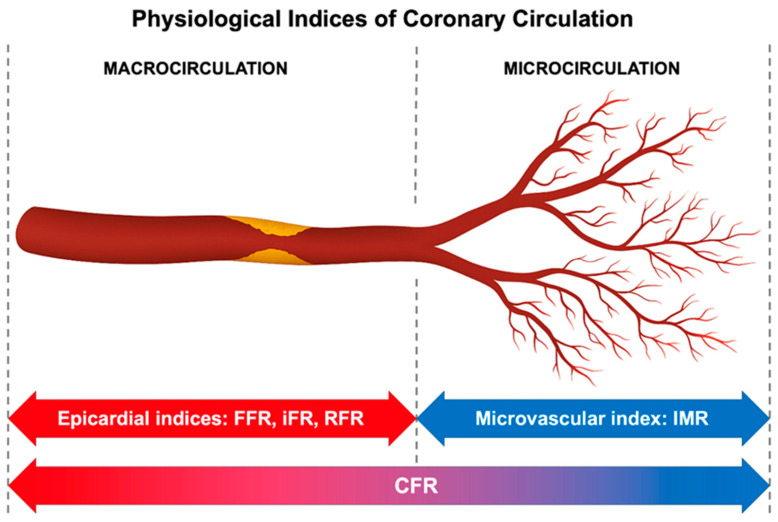
Physiological indices of coronary circulation. Schematic representation of the coronary circulation demonstrating the anatomical scope of physiological indices. The epicardial vessel (macrocirculation) contains a focal stenosis (yellow) and branches into the microvascular network (<400 μm diameter vessels). Epicardial indices (FFR, iFR, RFR) assess hemodynamic significance of epicardial stenoses. IMR specifically evaluates microvascular resistance. CFR provides integrated assessment across both compartments, quantifying overall coronary vasodilatory capacity. The gradient arrow for CFR illustrates its comprehensive evaluation from proximal epicardial to distal microvascular territories. FFR, fractional flow reserve; iFR, instantaneous wave-free ratio; RFR, resting full-cycle ratio; CFR, coronary flow reserve; IMR, index of microcirculatory resistance.

### 3.2. Invasive Assessment of Coronary Flow Reserve: Principles and Techniques (Figure 2)

Invasive techniques performed in the catheterization laboratory are foundational, though non-invasive quantification with PET is considered the gold standard for validation [57].

#### 3.2.1. Pharmacologic Induction of Hyperemia

Accurate assessment depends on inducing true maximal coronary hyperemia to minimize microcirculatory resistance [59] (Table 1).

The most widely used “gold standard” agent is adenosine, acting via adenosine A2 receptors [59]. It can be administered intravenously (IV; stable, steady-state hyperemia but more side effects) or intracoronary (IC; rapid, fewer systemic effects but transient hyperemia) [60,61,62,63].

Other agents that can be used include regadenoson (single IV bolus), adenosine triphosphate (ATP; equivalent to adenosine), and papaverine (older, risk of arrhythmias) [59,64]. The limitations of pharmacological hyperemia have spurred the development of non-hyperemic pressure ratios (NHPRs) like iFR and RFR [63] to assess clinically significant obstructive CAD.

**Figure 2:**
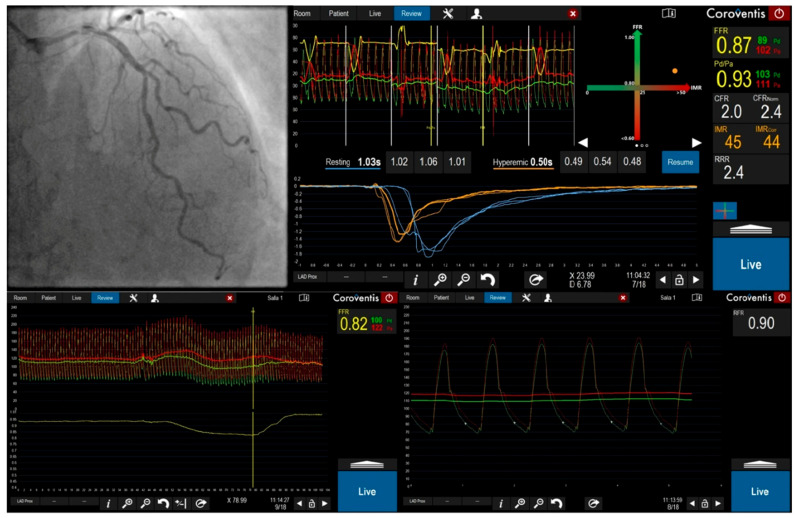
Invasive coronary physiological assessment demonstrating microvascular dysfunction in a symptomatic patient without obstructive coronary artery disease. A 69-year-old female with hypertension, active smoking, and CCS class II angina underwent comprehensive invasive physiological assessment of the left anterior descending artery. Coronary angiogram (**upper-left panel**) demonstrates diffuse atherosclerotic changes. Resting full-cycle ratio (RFR) was 0.90 and fractional flow reserve (FFR) during maximal hyperemia was 0.82 (lower panels). Both RFR and FFR values exceed ischemic thresholds (≤0.89 and ≤0.80, respectively), excluding hemodynamically significant epicardial stenosis. Thermodilution-derived indices (**upper-right panel**) reveal a coronary flow reserve (CFR) of 2.4 and index of microcirculatory resistance (IMR) of 44 U. The reduced CFR (<2.5) and elevated IMR (>25 U) indicate impaired coronary microvascular function, which likely accounts for the patient’s anginal symptoms despite non-obstructive epicardial disease.

#### 3.2.2. Doppler Guidewire-Based Technique (CFVR)

This method uses a guidewire with a Doppler transducer to measure flow velocity [65]. Coronary flow velocity reserve (CFVR) is calculated as the ratio of hyperemic to resting average peak velocity (APV). CFVR < 2.0 indicates hemodynamic significance [56,59,65]. Limitations include technical difficulty and operator dependence [53,66]. Combined sensor guidewires (pressure + Doppler) and emerging AI platforms for signal analysis aim to overcome these challenges (Table 2). Throughout this manuscript, ‘CFR’ is used as the general term for coronary flow reserve, while ‘CFVR’ (coronary flow velocity reserve) specifically denotes Doppler-derived velocity ratios.

#### 3.2.3. Thermodilution-Based Technique (CFRthermo)

This widely adopted alternative uses a pressure/temperature wire [55,67]. Coronary flow is derived by measuring the mean transit time (Tmn) of a saline bolus [68]. CFRthermo is calculated as Tmn(rest)/Tmn(hyperemia) [68]. The bolus method is standard [68], while continuous infusion allows the quantification of absolute coronary blood flow and demonstrates superior precision [69,70,71,72,73]. Thermodilution represents a pragmatic compromise, showing good correlation with Doppler but more modest correlation with PET [58,74].

### 3.3. A Comparative Framework: CFR, FFR, IMR, and Non-Hyperemic Indices

Modern laboratories use multiple indices for comprehensive assessment. Fractional Flow reserve (FFR) is a pressure-derived ratio (Pd/Pa during hyperemia) specific to epicardial stenosis [56,57,61]. Τhe threshold for detecting severe epicardial stenosis is a value ≤ 0.80 [59,75,76]. Coronary flow reserve (CFR) elaborates an integrated assessment of the entire pathway, evaluating both epicardial vessel and microcirculation. The threshold is <2.0 or <2.5, respectively [53,59,66]. Moreover, dedicated microvascular assessment can be performed by the index of microcirculatory resistance (IMR). IMR is derived from thermodilution method (Pd × Tmn during hyperemia) [77,78,79] and the threshold to detect microvascular dysfunction is ≥25 units [80,81,82]. Also, the measurement of non-hyperemic pressure ratios (iFR, RFR, dPR) is feasible. This method can evaluate epicardial stenosis at rest, avoiding adenosine. The threshold to diagnose severe epicardial stenosis is ≤0.89 [59,66]. Otherwise, a Pullback Pressure Gradient (PPG) may quantify CAD patterns (focal vs. diffuse) during PCI planning [83].

### 3.4. Integrated Assessment and CFR-IMR Discordance

Combining indices allows refined phenotyping [84,85]. Concordant abnormal values, characterized by low CFR and high IMR, indicate structural coronary microvascular dysfunction, which carries the highest risk for major adverse cardiovascular events. When CFR is low, but IMR remains normal, this pattern reflects functional CMD, a condition still associated with increased cardiovascular risk. In contrast, normal CFR combined with elevated IMR suggests compensated CMD, where the overall risk profile is similar to normal coronary physiology. Additionally, CFR measurement helps clarify mechanisms underlying discordance between FFR and iFR assessments [59,83,86,87].

### 3.5. Safety, Economic, and Guideline Considerations

Guidewire-based measurements carry low risk (0.5–2% complication rate) [59,67,81]. While FFR-guided PCI is cost-effective in stable CAD [59,66], comprehensive assessment (FFR, CFR, IMR) may prevent redundant testing in complex INOCA patients. The 2024 ESC Guidelines for Chronic Coronary Syndromes give a Class I recommendation for comprehensive invasive functional assessment (CFR, IMR, acetylcholine testing) in patients with suspected INOCA to enable precise endotyping and tailored therapy [88].

## 4. Non-Invasive Imaging Modalities for CFR Assessment

### 4.1. Transthoracic Doppler Echocardiography (TTDE)

**Figure 3:**
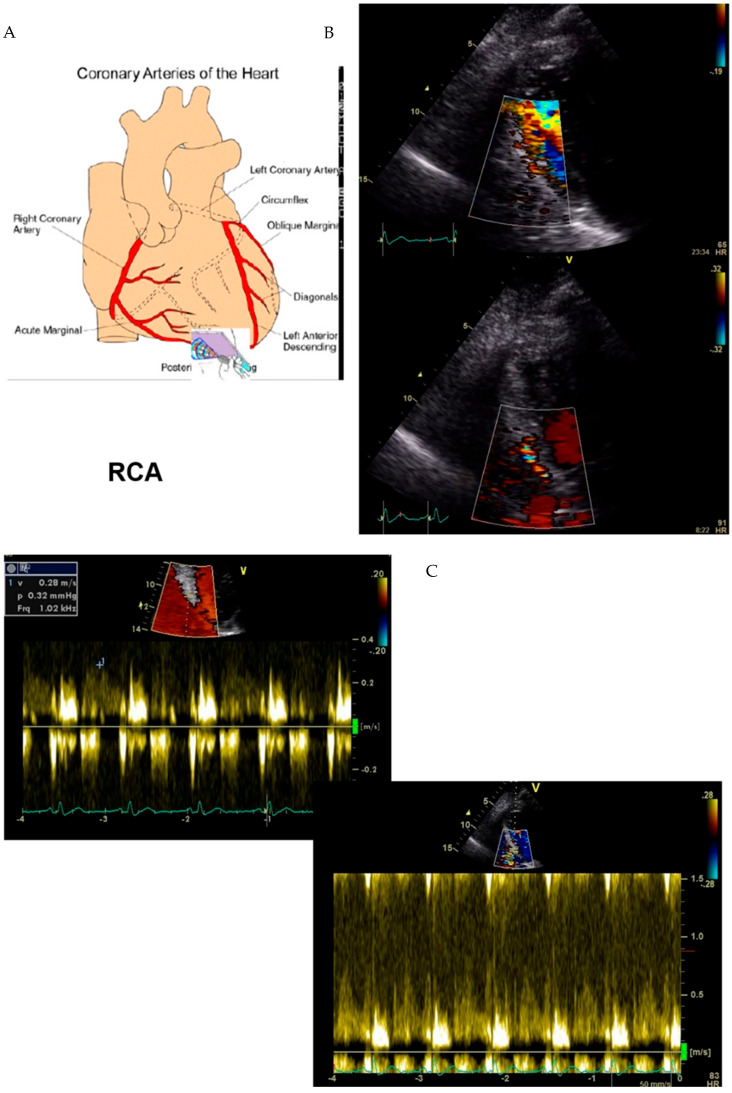
(**A**) Anatomical diagram of the coronary arteries, highlighting the course of the right coronary artery (RCA) and its major branches, including the acute marginal and posterior descending arteries. The illustration provides anatomical context for the coronary territory assessed in the accompanying ultrasound images. (**B**) Color Doppler transthoracic echocardiography image demonstrating blood flow within the RCA territory. The color flow map visualizes direction and velocity of coronary perfusion during the cardiac cycle. (**C**) Spectral Doppler waveform of the RCA, showing coronary flow velocity patterns across multiple cardiac cycles. Distinct systolic and diastolic components are visible, allowing assessment of coronary flow dynamics.

TTDE evaluates coronary blood flow velocity non-invasively, most commonly in the distal LAD [89], but CFR assessment is also feasible in the right coronary artery (Figure 3) and more seldom in circumflex artery. CFR is calculated as the ratio of peak hyperemic to resting diastolic flow velocities (CFVR). TTDE has detailed prognostic value, does not expose patients to radiation, is widely available and is cost effective. These features determine its value in clinical practice and make it suitable for serial evaluation [2,90]. While transthoracic Doppler echocardiography offers valuable non-invasive assessment of coronary flow reserve, its effectiveness is highly dependent on the skill and experience of the operator. The technique requires a significant learning curve. Additionally, its application is primarily limited to evaluating the left anterior descending (LAD) artery, with successful visualization achievable in approximately 85–90% of patients [2]. TTDE provides a velocity ratio rather than absolute flow and it depends on hemodynamics [91]. TTDE has merit in assessing microvascular dysfunction, evaluating intermediate macrovascular lesions in LAD pre- and post-intervention [92,93].

### 4.2. Positron Emission Tomography (PET)

Positron emission tomography (PET) stands as the reference non-invasive technique for quantitative assessment of myocardial blood flow (MBF) and coronary flow reserve (CFR), utilizing tracers such as ^13^N-ammonia, ^15^O-water, or ^82^Rb. While often referred to as the ‘gold standard’ for non-invasive CFR quantification, it should be acknowledged that this designation depends on local expertise, tracer availability, and appropriate infrastructure. One of the major strengths of PET is its ability to provide absolute quantification of MBF in milliliters per gram per minute, ensuring high reproducibility and robust prognostic value. This modality is particularly valuable because it can distinguish between epicardial and microvascular dysfunction, offering nuanced insights into coronary pathology. However, PET does have limitations, notably the use of ionizing radiation and the requirement for costly, specialized infrastructure, which may restrict its accessibility in certain clinical settings. Many centers lack access to PET or have limited tracer options, which may affect generalizability. Despite these challenges, PET is widely regarded as the state-of-the-art approach for risk stratification in patients with suspected coronary artery disease (CAD) and ischemia with no obstructive coronary arteries (INOCA), and it is strongly supported by current clinical guidelines. Importantly, emerging simplified protocols—such as those employing ^18^F-flurpiridaz—are anticipated to enhance accessibility and broaden the clinical utility of PET in the near future [94,95,96].

### 4.3. Cardiac Magnetic Resonance Imaging (CMR)

Cardiac magnetic resonance (CMR) perfusion imaging employs gadolinium-based contrast agents during both the rest and stress phases, allowing for quantitative analysis that yields measurements of myocardial blood flow (MBF) and myocardial perfusion reserve (MPR) [97]. This technique offers several notable advantages, including the absence of ionizing radiation, high spatial resolution, and the ability to provide multiparametric tissue characterization. Additionally, there is a growing body of prognostic evidence supporting its clinical value [98]. However, CMR perfusion imaging also presents certain limitations: it requires the use of a contrast agent, involves technical complexity, and may pose challenges for patient tolerance—such as the need for breath-holding and the potential for claustrophobia—as well as higher associated costs. Clinically, CMR is emerging as a valuable alternative to PET, with standardized quantitative protocols currently under development [99]. It is particularly useful for comprehensive tissue assessment in conjunction with perfusion analysis [100].

### 4.4. CT Perfusion and Dynamic SPECT

CT Perfusion (CTP) is a sophisticated imaging technique that merges anatomical information obtained from coronary computed tomography angiography (CTA) with functional data reflecting myocardial perfusion [101]. This dual capability allows clinicians to simultaneously visualize coronary artery anatomy and assess the physiological significance of detected lesions. The principal advantages of CTP lie in its ability to provide a comprehensive evaluation within a single imaging session and its high spatial resolution, which enhances the detection and characterization of coronary artery disease. However, CTP is not without limitations. The technique exposes patients to ionizing radiation and requires the administration of iodinated contrast agents, which may not be suitable for all individuals, particularly those with renal impairment or contrast allergies. Additionally, there is less standardization in the quantification of absolute myocardial blood flow compared to other modalities, which can affect the consistency and reliability of results across different centers. In clinical practice, CTP is primarily utilized to determine the functional significance of anatomical lesions identified on CTA, helping to guide management decisions regarding revascularization or medical therapy [101,102,103].

Dynamic SPECT, on the other hand, leverages advancements in detector technology—specifically the use of cadmium-zinc-telluride (CZT) detectors—to enable the kinetic modeling of myocardial blood flow (MBF) and the estimation of flow reserve [104]. The key strengths of dynamic SPECT include its broader availability and lower cost relative to positron emission tomography (PET), making it a pragmatic alternative in settings where PET or cardiac magnetic resonance (CMR) are not accessible. Despite these advantages, dynamic SPECT is generally considered to have inferior accuracy and reproducibility compared to PET and quantitative CMR. This limitation is particularly relevant when a precise quantification of myocardial blood flow is required for nuanced clinical decision-making. Nevertheless, dynamic SPECT serves an important role in expanding access to physiological assessment of coronary circulation, especially in resource-limited environments [105,106].

When selecting an imaging modality for the assessment of coronary flow reserve, clinicians must carefully weigh several trade-offs. In terms of accuracy and the ability to provide quantitative data, PET stands at the forefront, followed by quantitative CMR, transthoracic Doppler echocardiography (TTDE), and finally dynamic SPECT and CTP. PET’s superiority in quantification is well established, but it is counterbalanced by considerations of safety and accessibility. TTDE and CMR are preferable from a safety perspective, as they do not involve exposure to ionizing radiation, whereas PET, SPECT, and CTP do carry this risk. Availability and cost are also critical factors; TTDE is typically the most accessible and cost-effective, followed by SPECT, CMR, CTP, and PET, which is often the most expensive and least widely available.

## 5. Prognostic Significance of Coronary Flow Reserve

### 5.1. CFR in Stable Coronary Artery Disease (Table 3)

In stable CAD, CFR offers independent and incremental prognostic value beyond stenosis severity or FFR. Epicardial obstruction accounts for only part of the ischemic burden; diffuse atherosclerosis, endothelial dysfunction, and microvascular abnormalities can also impair coronary flow [10]. Impaired CFR predicts higher rates of all-cause mortality, cardiac death, and MACEs [107]. It can refine revascularization decisions, with evidence suggesting patients with impaired CFR may benefit more from PCI, while those with preserved CFR have a favorable prognosis with medical therapy alone [108,109,110].

**Table 3:** Prognostic studies of CFR in stable coronary artery disease (CAD).

Study/Year	Population	Method of CFR Assessment	Key Findings	Prognostic Outcome
Gould et al., 2007	2783 pts, suspected CAD	PET	CFR < 2 predicted mortality independent of stenosis	↑ CV death, MI
Johnson et al., 2012	1218 pts, stable CAD	Invasive Doppler/thermodilution	CFR discordant with FFR identified high-risk patients	↑ MACEs
Cortigiani et al., 2014	1280 pts, stress echo	Doppler echo	CFR < 2 doubled cardiac death risk	↑ Cardiac death
D’Antonio et al., 2025	Systematic review, PET	PET	Reduced CFR predicted mortality across studies	↑ All-cause death
Lee et al., 2015	1192 pts, revascularization decision	Invasive	CFR impaired → more benefit from PCI	↓ MACEs after PCI

CFR = Coronary Flow Reserve; CAD = Coronary Artery Disease; PET = Positron Emission Tomography; CV = Cardiovascular; MI = Myocardial Infarction; FFR = Fractional Flow Reserve; MACEs = Major Adverse Cardiovascular Events; PCI = Percutaneous Coronary Intervention.

### 5.2. CFR in Non-Obstructive CAD and INOCA (Table 4)

**Figure 4:**
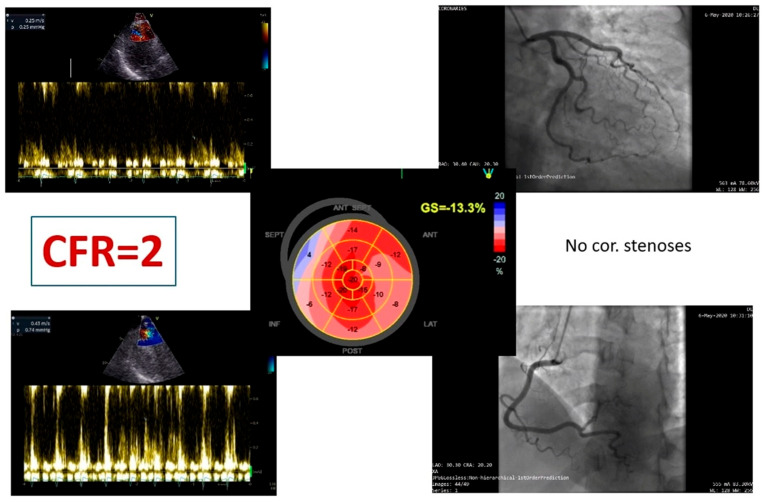
Coronary Microvascular Assessment Using Transthoracic Doppler Echocardiography and Coronary Angiography. (**Left panels**): Transthoracic Doppler echocardiography of the left anterior descending artery (LAD) at rest (**upper-left**) and during hyperemia (**lower-left**), demonstrating diastolic-dominant coronary flow used for calculation of coronary flow reserve (CFR). (**Right panels**): Coronary angiography showing no obstructive epicardial coronary stenoses. The central polar strain map shows reduced global longitudinal strain (GLS = 13.3%). The combined Doppler recordings yield a CFR = 2, consistent with microvascular dysfunction despite angiographically normal coronary arteries.

**Figure 5:**
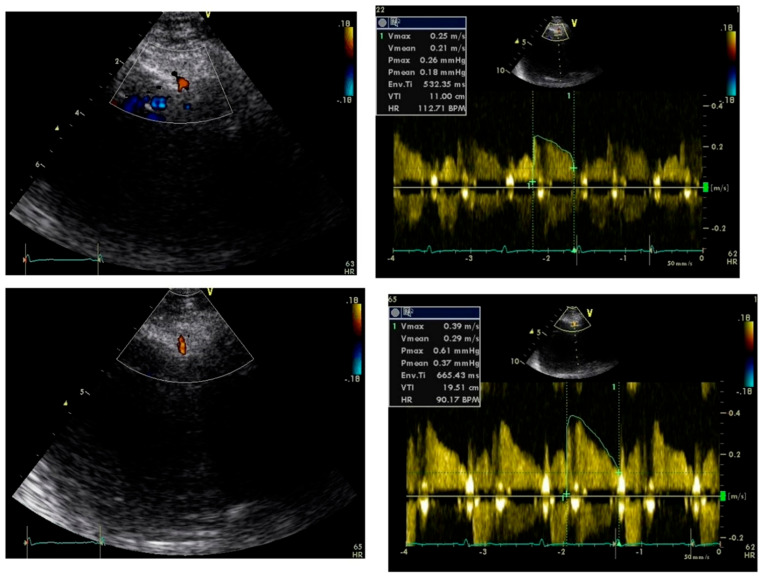
Coronary microvascular assessment using transthoracic Doppler. Transthoracic Doppler echocardiography of the left anterior descending artery (LAD) at rest (**upper-left**) and during hyperemia (**lower-left**), demonstrating diastolic-dominant coronary flow velocity patterns. The combined Doppler recordings yield a CFR < 2, consistent with significant stenosis of the left anterior descending artery.

INOCA, once considered benign, carries substantial morbidity and cardiovascular risk [7,111]. Impaired CFR is a central prognostic marker in INOCA, strongly associated with MACEs, mortality, and progression to HFpEF [7,10,112,113,114]. Women are disproportionately affected [30,31,115]. CFR assessment, now a Class I guideline recommendation [116,117,118], confirms diagnosis of INOCA and permits a tailored treatment to improve symptoms and quality of life [119] (Figure 4 and Figure 5).

**Table 4:** Prognostic implications of CFR in INOCA (Ischemia with No Obstructive Coronary Arteries).

Study/Year	Population	Sex Distribution	CFR Technique	Main Findings
Pepine et al., 2010	189 women, suspected ischemia	100% women	Invasive	Reduced CFR predicted higher MACEs
Murthy et al., 2014	1218 pts, mixed	~60% women	PET	CFR < 2 predicted death independent of CAD
Jensen et al., 2023	Meta-analysis, non-obstructive CAD	Mixed	Various (PET, CMR, Echo)	Impaired CFR strongly prognostic, stronger in women
Taqueti et al., 2018	329 women	100% women	PET	CFR < 2 → ↑ risk of HFpEF
Szolc et al., 2025	325 pts with INOCA	72% women	Invasive	CFR-based tailored therapy improved QoL

CFR = Coronary Flow Reserve; MACEs = Major Adverse Cardiovascular Events; PET = Positron Emission Tomography; CAD = Coronary Artery Disease; CMR = Cardiac Magnetic Resonance; HFpEF = Heart Failure with Preserved Ejection Fraction; INOCA = Ischemia with Non-Obstructive Coronary Arteries; QoL = Quality of Life.

### 5.3. CFR in Heart Failure and Cardiomyopathies (Table 5)

**Figure 6:**
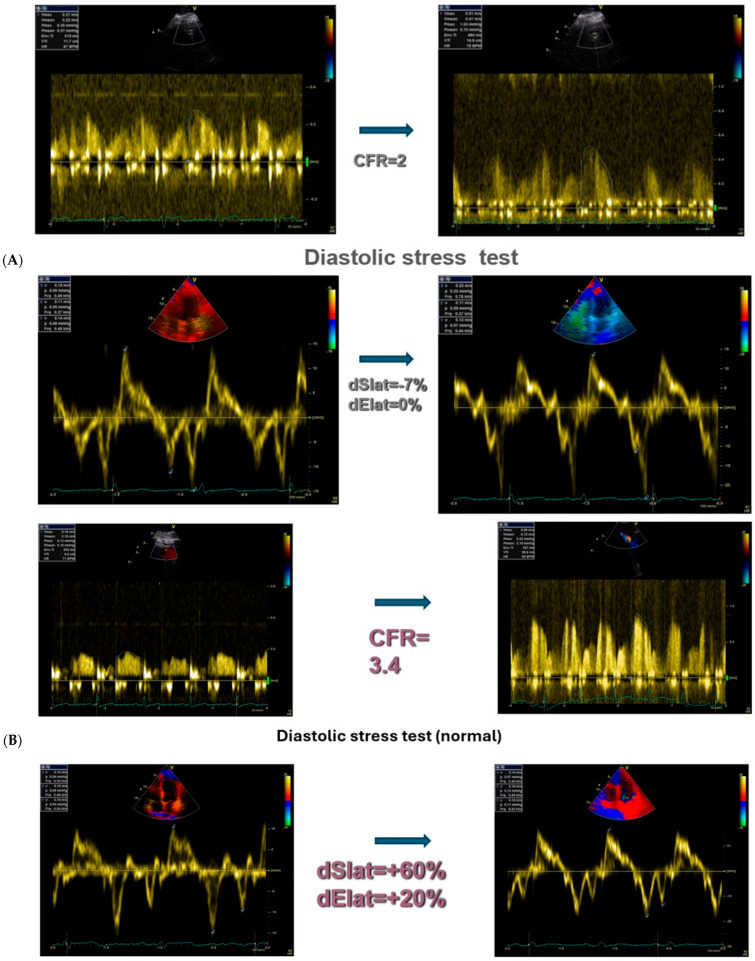
(**A**) Diastolic stress test—reduced coronary flow reserve. This panel illustrates Doppler-derived coronary flow velocities and tissue Doppler indices at rest and during stress. (**A**) Reduced coronary flow reserve (CFR = 2) indicating impaired vasodilatory response consistent with microvascular dysfunction. Tissue Doppler indices show limited augmentation of diastolic flow during stress. (**B**) Normal response (CFR = 3.4) demonstrating appropriate hyperemic augmentation. Tissue Doppler indices show preserved diastolic reserve (60% increase in septal e’ velocity, 20% increase in lateral e’ velocity).

In the context of HFpEF, coronary microvascular dysfunction and a reduction in CFR are increasingly recognized as central drivers of the underlying pathophysiology [14,120]. The impairment of microvascular function leads to an inadequate increase in coronary blood flow during periods of heightened myocardial demand, which in turn contributes to myocardial ischemia, diastolic dysfunction, and clinical manifestations of HFpEF. Numerous studies have demonstrated that a reduced CFR is not only prevalent in patients with HFpEF but also serves as an independent predictor of both the development of this condition and the risk of subsequent hospitalization. This highlights the importance of assessing CFR in individuals at risk for HFpEF, as early identification of microvascular dysfunction may allow for more targeted preventive and therapeutic strategies [121] (Figure 6).

**Table 5:** Prognostic role of CFR in heart failure and cardiomyopathy.

Study/Year	Population	Condition	CFR Assessment	Main Findings
Shah et al., 2016	244 pts with HFpEF	HFpEF	PET	Impaired CFR linked to diastolic dysfunction
Taqueti et al., 2018	329 women	HFpEF risk	PET	Reduced CFR predicted HFpEF development
Neglia et al., 2015	201 pts	Dilated cardiomyopathy	PET	CFR < 2 predicted mortality & hospitalization
Sciagrà et al., 2016	178 pts	Ischemic & non-ischemic cardiomyopathy	PET	Impaired CFR predicted MACEs
Toya et al., 2025	412 pts with HF	HFpEF & HFrEF	Invasive	CFR + high resistance → worse prognosis

CFR = Coronary Flow Reserve; PET = Positron Emission Tomography; HFpEF = Heart Failure with Preserved Ejection Fraction; HFrEF = Heart Failure with Reduced Ejection Fraction; MACEs = Major Adverse Cardiovascular Events.

Turning to HFrEF and cardiomyopathies, the impairment of CFR in these populations reflects the presence of diffuse microvascular dysfunction and adverse cardiac remodeling. In HFrEF, the inability of the coronary circulation to adequately augment blood flow in response to increased metabolic demands exacerbates myocardial injury and promotes the progression of heart failure. Similarly, in various forms of cardiomyopathy, reduced CFR is indicative of widespread microvascular disease and structural changes within the myocardium. The prognostic implications are significant: impaired CFR in these patients is associated with an increased risk of mortality, more frequent hospitalizations, and a heightened likelihood of arrhythmic events. Thus, CFR assessment provides valuable prognostic information and may inform risk stratification and management decisions in patients with HFrEF and cardiomyopathies [122,123,124].

### 5.4. CFR in Systemic Diseases (Table 6)

CFR has emerged as a powerful indicator of vascular health that extends beyond coronary artery disease. In the context of diabetes, CFR provides prognostic information that is independent of the severity of coronary artery disease or the degree of glycemic control. This means that even in patients whose coronary arteries do not show significant obstruction, a reduced CFR can still predict an increased risk of cardiovascular mortality. The ability of CFR to identify high-risk individuals among diabetic patients underscores its value in guiding early intervention and more aggressive management strategies [125,126].

**Table 6:** Prognostic significance of CFR in systemic diseases.

Disease	Key Study	Method	Main Findings	Prognostic Outcomes
Diabetes	Murthy et al., 2012	PET	CFR < 2 predicted CV death independent of CAD	↑ Mortality
Diabetes	Zhou et al., 2020	PET	CFR predicted outcomes independent of HbA1c	↑ CV events
CKD	Charytan et al., 2013	PET	CFR predicted CV mortality	↑ CV death
CKD	Shah et al., 2019	PET	CFR superior to eGFR in predicting outcomes	↑ HF hospitalization
SLE	Schindler et al., 2020	PET	Reduced CFR → ↑ CV risk in lupus	↑ MACEs
RA	Baniaamam et al., 2021	Echo Doppler	Impaired CFR predicted CV and all-cause death	↑ Mortality

CFR = Coronary Flow Reserve; PET = Positron Emission Tomography; CV = Cardiovascular; CAD = Coronary Artery Disease; HbA1c = Hemoglobin A1c; eGFR = Estimated Glomerular Filtration Rate; HF = Heart Failure; MACEs = Major Adverse Cardiovascular Events.

For individuals with chronic kidney disease, impairment of CFR has similarly profound implications. Studies have shown that reduced CFR in CKD patients is a strong predictor of cardiovascular mortality; in some cases, it may even surpass traditional markers—such as estimated glomerular filtration rate (eGFR)—in prognostic significance. This highlights the systemic nature of vascular dysfunction in CKD and the importance of CFR assessment in risk stratification and clinical decision-making for these patients [127,128].

Autoimmune diseases, including systemic lupus erythematosus and rheumatoid arthritis, are also associated with reduced CFR. In these populations, diminished CFR correlates with a higher incidence of cardiovascular events and increased mortality. The chronic inflammatory state characteristic of these conditions contributes to microvascular dysfunction, which is effectively captured by CFR measurement. As a result, CFR serves not only as a marker of coronary health but also as a window into the broader vascular consequences of systemic autoimmune disease [129,130].

Taken together, these findings illustrate that CFR is much more than a tool for evaluating coronary artery disease; it is a systemic biomarker that reflects the overall health of the vascular system. Its strong prognostic value in diabetes, CKD, and autoimmune diseases suggests potential for integration into clinical risk assessment. However, routine implementation requires careful consideration of local availability, cost, expertise, and—most importantly—whether CFR testing changes management decisions in ways that improve patient outcomes. Prospective studies demonstrating the clinical utility of CFR-guided management in these populations are needed before broad implementation can be recommended.

### 5.5. CFR in Guiding Therapy (Table 7)

CFR is increasingly recognized not only as a marker for risk stratification but also as a valuable tool for guiding therapeutic decisions. However, it is important to acknowledge that diagnostic phenotyping currently outpaces the evidence base for phenotype-specific treatments, particularly in CMD and INOCA.

**Table 7:** Clinical utility of CFR in guiding therapy.

Strategy	Key Study	Population	CFR Finding	Clinical Implication
Revascularization	Lee et al., 2015	1192 pts, stable CAD	Impaired CFR → benefit from PCI	CFR can stratify who benefits most
Medical therapy	Bairey Merz et al., 2019	189 women with microvascular angina	Ranolazine improved CFR & angina	Tailored pharmacotherapy
Risk factor control	Takx et al., 2016	Mixed CAD cohorts	Statins/ACEi improved CFR	Preventive therapy enhances CFR
Lifestyle interventions	Mehta et al., 2021	Women with CMD	Exercise & lifestyle improved CFR	Non-pharmacologic therapy
Clinical trials	Al-Gully et al., 2025	INOCA & CAD pts	Discordant CFR & resistance predicted outcomes	CFR as a trial endpoint

CFR = Coronary Flow Reserve; ACEi = Angiotensin-Converting Enzyme Inhibitor; PCI = Percutaneous Coronary Intervention; CAD = Coronary Artery Disease; CMD = Coronary Microvascular Dysfunction; INOCA = Ischemia with Non-Obstructive Coronary Arteries.

Revascularization: CFR assessment can help determine which patients with intermediate coronary lesions are most likely to benefit from percutaneous coronary intervention. Evidence suggests that patients with impaired CFR derive greater benefit from PCI, whereas those with preserved CFR have favorable outcomes with medical therapy alone [131]. However, this evidence derives largely from observational or post hoc analyses; prospective trials using CFR to guide revascularization decisions are lacking.

Pharmacological therapy: Several interventions have demonstrated the ability to improve CFR, including statins, ACE inhibitors, and ranolazine [132,133,134]. However, most studies have focused on surrogate endpoints (improvement in CFR or angina symptoms) rather than hard clinical outcomes such as mortality or MACE reduction. The evidence for ranolazine, for example, shows improvement in angina and CFR but inconsistent effects on hospitalization or major events. Similarly, while risk factor modification (glycemic control, blood pressure reduction, lipid lowering) and lifestyle interventions have been associated with improved CFR in observational studies [135,136], high-quality randomized trials demonstrating that CFR-guided therapy improves outcomes are still needed.

Clinical trials: CFR is increasingly used as a surrogate endpoint in clinical trials [84,137,138]. While this reflects its biological plausibility and responsiveness to interventions, investigators and clinicians should recognize that improvement in CFR does not necessarily guarantee improvement in patient-important outcomes. The field awaits large-scale randomized controlled trials using CFR as a selection tool or target to demonstrate improvements in hard endpoints.

### 5.6. Emerging Role of CFR in Arrhythmogenic Conditions and Epicardial Disease

Beyond the conventional contexts of CAD, INOCA, and heart failure, CFR may also have relevance in clinical scenarios characterized by epicardial abnormalities and arrhythmogenic substrates. The interaction between epicardial tissue pathology, coronary microvascular function, and myocardial perfusion may carry diagnostic and prognostic implications in patients with inherited arrhythmogenic syndromes. For example, in Brugada syndrome—a condition associated with an increased risk of ventricular arrhythmias and sudden cardiac death—epicardial substrate characterization has emerged as an important area of investigation. Advanced procedural approaches, including epicardial ablation, are increasingly used to modify arrhythmogenic substrates in these patients [139]. The potential relationship between coronary microvascular dysfunction, as assessed by CFR, and the severity or inducibility of arrhythmogenic substrates has not been systematically investigated. However, given that myocardial ischemia and microvascular abnormalities can create arrhythmogenic conditions through mechanisms including altered conduction velocity, increased dispersion of repolarization, and sympathetic dysregulation, CFR assessment might theoretically provide complementary information in the risk stratification of selected patients with epicardial arrhythmogenic disorders. Future studies are warranted to explore whether CFR adds value to current arrhythmia risk prediction models or helps guide procedural planning in patients undergoing epicardial substrate ablation.

## 6. Future Directions and Emerging Technologies

### 6.1. Advancements in Non-Invasive and Hybrid Imaging

PET/CT and PET/MR hybrid imaging combine functional perfusion data with high-resolution anatomy (CT) or superior tissue characterization (MR), advancing toward personalized, biologically guided management [78,88,140].

### 6.2. Artificial Intelligence and Advanced Computational Approaches

Artificial intelligence (AI) is also revolutionizing the derivation of physiological insights from anatomical imaging. AI-powered analysis of coronary computed tomography angiography (CTA) enables the non-invasive computation of fractional flow reserve (FFR-CT). This technology has expanded the role of physiological assessment by allowing clinicians to determine the functional significance of coronary lesions without the need for invasive pressure wires or pharmacological stress. FFR-CT is particularly valuable for patient selection prior to invasive angiography, helping to avoid unnecessary procedures in patients without hemodynamically significant stenoses. Emerging applications include the evaluation of patients with prior coronary stents, where traditional CTA is often limited by blooming artifact; preliminary studies suggest FFR-CT may have utility in this challenging population [140]. However, current evidence remains limited, and the technique requires further validation in complex coronary anatomy, including diffuse disease, tandem lesions, and vessels with heavy calcification.

Beyond the image-based computation of FFR, AI is increasingly being integrated into invasive cardiovascular diagnostics to support real-time decision-making. Machine learning algorithms can now automate the interpretation of pressure and flow signals during catheterization procedures, reducing operator dependence and improving measurement consistency. AI-based approaches have been developed to optimize wire-based physiological measurements by identifying artifacts, predicting optimal hyperemic conditions, and detecting discordant patterns among CFR, FFR, and IMR that may indicate specific pathophysiological phenotypes. Furthermore, AI is enabling the integration of multimodal data—combining pressure, flow, imaging, and electrophysiological signals—during complex procedures such as electrophysiology catheterization, where the interaction between arrhythmogenic substrates and coronary microvascular function may have diagnostic and prognostic implications [141].

Despite these advances, caution is warranted. Variable results from clinical trials, such as FAVOR III Europe, indicate that angiography-based FFR and QFR are not yet validated replacements for traditional wire-based indices when making decisions about deferring interventions. Continued research and refinement are needed before these AI-driven methods can be fully integrated into routine clinical practice [142].

### 6.3. Closing the Diagnosis–Treatment Gap

The major challenge ahead is translating diagnostic advances into proven therapies. Accurate endotyping of CMD via CFR and related indices has identified distinct pathophysiological phenotypes (structural CMD, functional CMD, compensated CMD), but targeted, phenotype-specific treatments validated in large-scale randomized controlled trials remain limited. The diagnosis–treatment gap is particularly evident in INOCA and CMD, where patients can now be precisely phenotyped using invasive or non-invasive CFR assessment, but evidence-based algorithms linking specific phenotypes to effective therapies are still emerging.

Current guideline recommendations (ESC 2024, Class I) support comprehensive invasive functional testing for suspected INOCA to enable precise endotyping. However, these recommendations are based largely on observational data and expert consensus rather than large, randomized outcome trials. Moving forward, the field requires: (1) randomized controlled trials of phenotype-specific treatments using hard clinical endpoints; (2) studies demonstrating that CFR-guided management improves outcomes compared with standard care; and (3) cost-effectiveness analyses to support implementation in diverse healthcare settings. Until such evidence accumulates, CFR should be viewed as a powerful prognostic tool and an emerging guide to therapy, rather than a fully validated treatment-directing biomarker.

## 7. Conclusions

The assessment and management of CAD have undergone a profound, guideline-codified transformation toward comprehensive physiological assessment. Coronary flow reserve has evolved from a research concept to an increasingly important clinical tool, providing indispensable integrated insight into coronary vascular health, robustly stratifying risk across cardiovascular and systemic conditions, and guiding personalized therapeutic decisions in selected patient populations. The future lies in the intelligent integration of multiple data streams—using non-invasive tools like FFR-CT as gatekeepers, complemented by invasive wire-based comprehensive assessment when needed—all supported by AI-driven efficiency. The ultimate goal is to leverage precise physiological phenotyping to close the diagnosis–treatment gap and deliver effective, tailored strategies for all manifestations of coronary disease.

## Figures and Tables

**Table 1:** Pharmacologic agents for induction of coronary hyperemia.

Agent	Route	Dosage	Half-Life	Time to Maximal Hyperemia	Advantages	Disadvantages
Adenosine	IV	140 μg/kg/min	1–2 min	<1–2 min	Gold standard; produces stable, steady-state hyperemia	Systemic side effects (hypotension, chest discomfort, dyspnea); requires central access; higher cost; time-consuming
Adenosine	IC	60–100 μg (LCA) 20–30 μg (RCA)	30–60 s	5–10 s	Rapid onset; brief duration; fewer systemic effects; lower cost	Transient hyperemia may be suboptimal; risk of transient AV block
Regadenoson	IV	0.4 mg bolus	2–4 min (functional effect ≤ 30 min)	1–4 min	Single bolus administration; A_2_a receptor selective	Prolonged action delays subsequent measurements; tachycardia; higher cost
ATP	IC	40 μg	Similar to adenosine	Similar to adenosine	Equivalent to adenosine for CFR/FFR measurements	Similar side effect profile to adenosine
Papaverine	IC	10–15 mg	2 min	20–60 s	Effective; short-acting	Risk of ventricular arrhythmias (torsades de pointes); hypotension

Abbreviations: IV, intravenous; IC, intracoronary; LCA, left coronary artery; RCA, right coronary artery; CFR, coronary flow reserve; FFR, fractional flow reserve; ATP, adenosine triphosphate; AV, atrioventricular.

**Table 2:** Comparison of invasive physiological indices.

Feature	CFR	FFR	iFR/RFR/dPR	IMR	HMR	HSR	RRR	PPG
Physiological principle	Ratio of hyperemic to resting flow	Ratio of distal to aortic pressure during hyperemia	Ratio of distal to aortic pressure at rest	Product of distal pressure and transit time during hyperemia	Ratio of distal pressure to flow velocity during hyperemia	Ratio of pressure gradient to flow velocity during hyperemia	Ratio of resting to hyperemic microvascular resistance	Longitudinal distribution of pressure loss
Primary application	Global vascular function; CMD diagnosis	Epicardial stenosis assessment	Epicardial stenosis assessment	Microvascular resistance (thermodilution-based)	Microvascular resistance (Doppler-based)	Epicardial stenosis assessment	Microvascular vasodilatory capacity	Focal versus diffuse disease pattern
Measurement technique	Doppler or thermodilution	Pressure wire	Pressure wire	Thermodilution wire	Doppler wire	Doppler wire	Doppler or thermodilution	Pressure wire pullback
Hyperemia required	Yes	Yes	No	Yes	Yes	Yes	Yes	Optional
Anatomical specificity	Global (epicardial + microvascular)	Epicardial	Epicardial	Microvascular	Microvascular	Epicardial	Microvascular	Epicardial pattern
Abnormal threshold	<2.0 (<2.5 depending on modality)	≤0.80	≤0.89	≥25 units	≥2.5 mmHg·cm^−1^·s	≥0.8 mmHg·cm^−1^·s	<2.62	0 (diffuse) to 1 (focal)

Abbreviations: CFR, coronary flow reserve; FFR, fractional flow reserve; iFR, instantaneous wave-free ratio; RFR, resting full-cycle ratio; IMR, index of microcirculatory resistance; HMR, hyperemic microvascular resistance; HSR, hyperemic stenosis resistance; RRR, resistive reserve ratio; PPG, pullback pressure gradient; CMD, coronary microvascular dysfunction; dPR, Diastolic Pressure Ratie. The abnormal threshold for CFR depends on the modality used.

## Data Availability

No new data were created or analyzed in this study.

## Abbreviations

CFR, coronary flow reserve; CFVR, coronary flow velocity reserve, mainly Doppler-derived; MBF, myocardial blood flow; MFR, myocardial flow reserve; MPR, myocardial perfusion reserve.

## References

[1] Hozumi T., Yoshida K., Ogata Y., Akasaka T., Asami Y., Takagi T., Morioka S. (1998). Noninvasive assessment of significant left anterior descending coronary artery stenosis by coronary flow velocity reserve with transthoracic color Doppler echocardiography. Circulation.

[2] Homuzi T., Yoshida K., Akasaka T., Asami Y., Ogata Y., Takagi T., Kaji S., Kawamoto T., Ueda Y., Morioka S. (1998). Noninvasive assessment of coronary flow velocity and coronary flow velocity reserve in the left anterior descending coronary artery by Doppler echocardiography: Comparison with invasive technique. J. Am. Coll. Cardiol..

[3] Gould K.L., Johnson N.P., Bateman T.M., Beanlands R.S., Bengel F.M., Bober R., Camici P.G., Cerqueira M.D., Chow B.J., Di Carli M.F. (2013). Anatomic versus physiologic assessment of coronary artery disease. J. Am. Coll. Cardiol..

[4] Youn H.-J., Foster E. (2004). Demonstration of coronary artery flow using transthoracic Doppler echocardiography. J. Am. Soc. Echocardiogr..

[5] Voci P., Pizzuto F., Romeo F. (2004). Coronary flow: A new asset for the echo lab?. Eur. Heart J..

[6] Fu B., Wei X., Lin Y., Chen J., Yu D. (2022). Pathophysiologic Basis and Diagnostic Approaches for Ischemia with Non-obstructive Coronary Arteries. Front. Cardiovasc. Med..

[7] Jensen S.M., Prescott E.I.B., Abdulla J. (2023). The prognostic value of coronary flow reserve in patients with non-obstructive coronary artery disease and microvascular dysfunction: A systematic review and meta-analysis with focus on imaging modality and sex difference. Int. J. Cardiovasc. Imaging.

[8] Galiuto L., Sestito A., Barchetta S., Sgueglia G.A., Infusino F., La Rosa C., Lanza G., Crea F. (2007). Noninvasive evaluation of flow reserve in the left anterior descending coronary artery in patients with cardiac syndrome X. Am. J. Cardiol..

[9] Egashira K., Inou T., Hirooka Y., Yamada A., Urabe Y., Takeshita A. (1993). Evidence of impaired endothelium-dependent coronary vasodilatation in patients with angina pectoris and normal coronary angiograms. N. Engl. J. Med..

[10] Camici P.G., Crea F. (2007). Coronary microvascular dysfunction. N. Engl. J. Med..

[11] Takeuchi M., Lodato J.A., Furlong K.T., Lang R.M., Yoshikawa J. (2005). Feasibility of measuring coronary flow velocity and reserve in the left anterior descending coronary artery by transthoracic Doppler echocardiography in the relatively obese American population. Echocardiography.

[12] Meimoun P., Clerc J., Ardourel D., Djou U., Martis S., Botoro T., Elmkies F., Zemir H., Luycx-Bore A., Boulanger J. (2017). Assessment of left anterior descending artery stenosis of intermediate severity by fractional flow reserve, instantaneous wave-free ratio, and non-invasive coronary flow reserve. Int. J. Cardiovasc. Imaging.

[13] Kasprzak J.D., Wejner-Mik P., Nouri A., Szymczyk E., Krzemińska-Pakuła M., Lipiec P. (2013). Transthoracic measurement of left coronary artery flow reserve improves the diagnostic value of routine dipyridamole-atropine stress echocardiogram. Arch. Med. Sci..

[14] Kelshiker M., Seligman H., Howard J., Haseeb Rahman H., Foley M., Nowbar A., Rajkumar C.A., Shun-Shin M.J., Ahmad Y., Sen S. (2022). Coronary flow reserve and cardiovascular outcomes: A systematic review and meta-analysis. Eur. Heart J..

[15] Tjoe B., Barsky L., Wei C., Samuels B., Azarbal B., Merz C., Shufelt C. (2022). Coronary microvascular dysfunction: Considerations for diagnosis and treatment. Clevel. Clin. J. Med..

[16] Bruning R., Sturek M. (2015). Benefits of exercise training on coronary blood flow in coronary artery disease patients. Prog. Cardiovasc. Dis..

[17] Ghodeshwar G., Dube A., Khobragade D. (2023). Impact of lifestyle modifications on cardiovascular health: A narrative review. Cureus.

[18] Bove K., Michelsen M., Schroder J., Suhrs H., Bechsgaard D., Mygind N., Aziz A., Kastrup J., Gustafsson I., Prescott E. (2021). Impaired coronary flow velocity reserve is associated with cardiovascular risk factors but not with angina symptoms. Open Heart.

[19] Padro T., Manfrini O., Bugiardini R., Canty J., Cenko E., De Luca G., Duncker D.J., Eringa E.C., Koller A., Tousoulis D. (2020). ESC Working Group on Coronary Pathophysiology and Microcirculation position paper on ‘coronary microvascular dysfunction in cardiovascular disease. Cardiovasc. Res..

[20] Shah S., Lam C., Svedlund S., Saraste A., Hage C., Tan R., Beussink-Nelson L., Ljung Faxén U., Fermer M.L., Broberg M.A. (2018). Prevalence and correlates of coronary microvascular dysfunction in heart failure with preserved ejection fraction: PROMIS-HFpE. Eur. Heart J..

[21] Shah S., Borlaug B., Kitzman D., McCulloch A., Blaxall B., Agarwal R., Chirinos J.A., Collins S., Deo R.C., Gladwin M.T. (2020). Research priorities for heart failure with preserved ejection fraction. Circulation.

[22] Donato A., Machin D., Lesniewski L. (2018). Mechanisms of Dysfunction in the Aging Vasculature and Role in Age-Related Disease. Circ. Res..

[23] Mátyás B.B., Benedek I., Rat N., Gerculy R., Benedek T. (2026). Coronary Plaque Vulnerability and Pericoronary Adipose Tissue Inflammation: Emerging Insights from Advanced CT Imaging. Medicina.

[24] Sperry B., Metzinger M., Ibrahim A., Thompson R., Cho Y., Jones P., McGhie A., Bateman T. (2024). Age-sex-specific myocardial blood flow values in patients without coronary atherosclerosis on Rb-82 PET myocardial perfusion imaging. Circ. Cardiovasc. Imaging.

[25] Faria D., Mejia-Renteria H., Lee J., Lee S., Travieso A., Jung J., Doh J., Nam C., Shin E., Hoshino M. (2022). Age-related changes in the coronary microcirculation influencing the diagnostic performance of invasive pressure-based indices and long-term patient prognosis. Catheter. Cardiovasc. Interv..

[26] Galderisi M., Rigo F., Gherardi S., Cortigiani L., Santoro C., Sicari R., Picano E. (2012). The impact of aging and atherosclerotic risk factors on transthoracic coronary flow reserve in subjects with normal coronary angiography. Cardiovasc. Ultrasound.

[27] Hoef T., Echavarria-Pinto M., Meuwissen M., Stegehuis V., Escaned J., Piek J. (2020). Contribution of age-related microvascular dysfunction to abnormal coronary: Hemodynamics in patients with ischemic heart disease. J. Am. Coll. Cardiol. Cardiovasc. Interv..

[28] Taqueti V., Di Carli M. (2018). Coronary microvascular disease pathogenic mechanisms and therapeutic options: State-of-the-art review. J. Am. Coll. Cardiol..

[29] Crea F., Battipaglia I., Andreotti F. (2015). Sex differences in mechanisms, presentation and management of ischaemic heart disease. Atherosclerosis.

[30] Murthy V., Naya M., Taqueti V., Foster C., Gaber M., Hainer J., Dorbala S., Blankstein R., Rimoldi O., Camici P.G. (2014). Effects of sex on coronary microvascular dysfunction and cardiac outcomes. Circulation.

[31] Pepine C., Anderson R., Sharaf B., Reis S., Smith K., Handberg E., Delia Johnson B., Sopko G., Noel Bairey Merz C. (2010). Coronary microvascular reactivity to adenosine predicts adverse outcome in women evaluated for suspected ischemia: Results from the NHLBI Women’s Ischemia Syndrome Evaluation (WISE). J. Am. Coll. Cardiol..

[32] Taqueti V. (2018). Sex Differences in the Coronary System. Adv. Exp. Med. Biol..

[33] Wei J., Mehta P., Johnson B., Samuels B., Kar S., Anderson R., Azarbal B., Petersen J., Sharaf B., Handberg E. (2012). Safety of coronary reactivity testing in women with no obstructive coronary artery disease: Results from the NHLBI-sponsored WISE (Women’s Ischemia Syndrome Evaluation) study. JACC Cardiovasc. Interv..

[34] Marinescu M., Löffler A., Ouellette M., Smith L., Bourque J. (2015). Coronary Microvascular Dysfunction, Microvascular Angina, and Treatment Strategies. JACC Cardiovasc. Interv..

[35] Taqueti V., Shaw L., Cook N., Murthy V., Shah N., Foster C., Hainer J., Blankstein R., Dorbala S., Di Carli M.F. (2017). Excess cardiovascular risk in women relative to men referred for coronary angiography is associated with severely impaired coronary flow reserve, not obstructive disease. Circulation.

[36] Yu Z., Jiao Y., Zhao Y., Gu W. (2022). Level of estrogen in females—The different impacts at different life stages. J. Pers. Med..

[37] Corban M., Prasad A., Gulati R., Lerman L., Lerman A. (2021). Sex-specific differences in coronary blood flow and flow velocity reserve in symptomatic patients with non-obstructive disease. EuroIntervention.

[38] Jalnapurkar S., Landes S., Wei J., Mehta P., Shufelt C., Minissian M., Pepine C.J., Handberg E., Cook-Wiens G., Sopko G. (2021). Coronary endothelial dysfunction appears to be a manifestation of a systemic process: A report from the Women’s Ischemia Syndrome Evaluation—Coronary Vascular Dysfunction (WISE-CVD) study. PLoS ONE.

[39] Patel K., Shaw L., Spertus J., Sperry B., McGhie A., Kennedy K., Thompson R.C., Chan P.S., Bateman T.M. (2022). Association of sex, reduced myocardial flow reserve, and long-term mortality across spectrum of atherosclerotic disease. JACC Cardiovasc. Interv..

[40] Houghton J., Prisant M., Carr A., Flowers N., Frank M. (1994). Racial differences in myocardial ischemia and coronary flow reserve in hypertension. J. Am. Coll. Cardiol..

[41] Zhang L., Olalere D., Mayrhofer T. (2022). Difference in cardiovascular risk, coronary artery disease, and cardiac events between black and white individuals enrolled in the PROMISE Trial. JAMA Cardiol..

[42] Breathett K., Sims M., Gross M., Jackson E., Jones E., Navas-Acien A., Taylor H., Thomas K.L., Howard B.V., On behalf of the American Heart Association Council on Epidemiology (2020). Cardiovascular health in American Indians & Alaska Natives: A scientific statement from the American Heart Association. Circulation.

[43] Gupta M., Singh N., Verma S. (2006). South Asians and cardiovascular risk: What clinicians should know. Circulation.

[44] Ihdayhid A., Thakur U., Yap G., Goeller M., Nerlekar N., Daniel Adams D., Isa M., Joshi M., Cameron J., Seneviratne S. (2021). Ethnic differences in coronary anatomy, left ventricular mass and CT-derived fractional flow reserve. J. Cardiovasc. Comput. Tomogr..

[45] Miller M., Cappuccio F. (2007). Ethnicity and Inflammatory Pathways—Implications for Vascular Disease, Vascular Risk and Therapeutic Intervention. Curr. Med. Chem..

[46] Tanus-Santos J.E., Desai M., Flockhart D.A. (2001). Effects of ethnicity on the distribution of clinically relevant endothelial nitric oxide variants. Pharmacogenetics.

[47] Shantsila E., Wrigley B., Shantsila A., Tapp L., Blann A., Gill P., Lip G. (2011). Ethnic differences in macrovascular and microvascular function in systolic heart failure. Circ. Heart Fail..

[48] Young A., Sullivan S., Moazzami K., Lima B., Shah A., Lewis T., Elon L., Li L., Quyyumi A., Vaccarino V. (2019). Abstract P137: Impaired microvascular and endothelial function among African-Americans with coronary artery disease. Circulation.

[49] AlJaroudi W., Hage F. (2018). Review of cardiovascular imaging in the Journal of Nuclear Cardiology 2017. Part 1 of 2: Positron emission tomography, computed tomography, and magnetic resonance. J. Nucl. Cardiol..

[50] Kaufmann P., Camici P. (2005). Myocardial blood flow measurement by PET: Technical aspects and clinical applications. J. Nucl. Med..

[51] Murthy V., Di Carli M. (2012). Non-invasive quantification of coronary vascular dysfunction for diagnosis and management of coronary artery disease. J. Nucl. Cardiol..

[52] Nesterov S., Deshayes E., Sciagrà R., Settimo L., Declerck J., Pan X., Yoshinaga K., Katoh C., Slomka P.J., Germano G. (2015). Quantification of myocardial blood flow in absolute terms using ^82^Rb PET imaging: The RUBY-10 Study. JACC Cardiovasc. Interv..

[53] Fearon W.F., Kobayashi Y. (2017). Invasive Assessment of the Coronary Microvasculature: The Index of Microcirculatory Resistance. Circ. Cardiovasc. Interv..

[54] Mangiacapra F., Viscusi M.M., Verolino G., Paolucci L., Nusca A., Melfi R., Ussia G.P., Grigioni F. (2021). Invasive Assessment of Coronary Microvascular Function. J. Clin. Med..

[55] Belmonte M., Gallinoro E., Pijls N.H.J., Bertolone D.T., Keulards D.C.J., Viscusi M.M., Storozhenko T., Mizukami T., Mahendiran T., Seki R. (2024). Measuring Absolute Coronary Flow and Microvascular Resistance by Thermodilution: JACC Review Topic of the Week. J. Am. Coll. Cardiol..

[56] Cokkinos D.V., Manginas A., Voudris V. (2003). Coronary flow: Clinical considerations. Heart.

[57] Ciaramella L., Di Serafino L., Mitrano L., De Rosa M.L., Carbone C., Rea F.S., Monaco S., Scalamogna M., Cirillo P., Esposito G. (2023). Invasive Assessment of Coronary Microcirculation: A State-of-the-Art Review. Diagnostics.

[58] Everaars H., de Waard G.A., Driessen R.S., Danad I., van de Ven P.M., Raijmakers P.G., Lammertsma A.A., van Rossum A.C., Knaapen P., van Royen N. (2018). Doppler Flow Velocity and Thermodilution to Assess Coronary Flow Reserve: A Head-to-Head Comparison with [^15^O]H_2_O PET. JACC Cardiovasc. Interv..

[59] Gurav A., Revaiah P.C., Tsai T.Y., Miyashita K., Tobe A., Oshima A., Sevestre E., Garg S., Aben J.P., Reiber J.H.C. (2024). Coronary angiography: A review of the state of the art and the evolution of angiography in cardio therapeutics. Front. Cardiovasc. Med..

[60] Layland J., Carrick D., Lee M., Oldroyd K., Berry C. (2014). Adenosine: Physiology, pharmacology, and clinical applications. JACC Cardiovasc. Interv..

[61] Sandhu P.S., Kaul U., Gupta R.K., Ghose T. (2013). Fractional flow reserve: Intracoronary versus intravenous adenosine induced maximal coronary hyperemia. Indian Heart J..

[62] Legutko J., Kleczyński P., Dziewierz A., Rzeszutko Ł., Dudek D. (2019). Adenosine intracoronary bolus dose escalation versus intravenous infusion to induce maximum coronary hyperemia for fractional flow reserve assessment. Kardiol. Pol..

[63] Zdzierak B., Zasada W., Krawczyk-Ożóg A., Rakowski T., Bartuś S., Surdacki A., Dziewierz A. (2023). Influence of sex on the functional assessment of myocardial ischemia. Kardiol. Pol..

[64] Jeremias A., Filardo S.D., Whitbourn R.J., Kernoff R.S., Yeung A.C., Fitzgerald P.J., Yock P.G. (2000). Effects of intravenous and intracoronary adenosine 5′-triphosphate as compared with adenosine on coronary flow and pressure dynamics. Circulation.

[65] Akasaka T., Yamamuro A., Kamiyama N., Koyama Y., Akiyama M., Watanabe N., Neishi Y., Takagi T., Shalman E., Barak C. (2003). Assessment of coronary flow reserve by coronary pressure measurement: Comparison with flow- or velocity-derived coronary flow reserve. J. Am. Coll. Cardiol..

[66] Petretta M., Acampa W., Zampella E., Assante R., Petretta M.P., Cuocolo R., Fabiani I., Della Rattal G.L., Perrone-Filardi P., Cuocolo A. (2011). Imaging techniques for assessment of coronary flow reserve. Monaldi Arch. Chest Dis..

[67] Barbato E., Aarnoudse W., Aengevaeren W.R., Werner G., Klauss V., Bojara W., Herzfeld I., Oldroyd K.G., Pijls N.H., De Bruyne B. (2004). Validation of coronary flow reserve measurements by thermodilution in clinical practice. Eur. Heart J..

[68] Candreva A., Gallinoro E., van’t Veer M., Sonck J., Collet C., Di Gioia G., Kodeboina M., Mizukami T., Nagumo S., Keulards D. (2021). Basics of Coronary Thermodilution. JACC Cardiovasc. Interv..

[69] De Bruyne B., Pijls N.H., Smith L., Wievegg M., Heyndrickx G.R. (2001). Coronary thermodilution to assess flow reserve: Experimental validation. Circulation.

[70] Gallinoro E., Bertolone D.T., Mizukami T., Paolisso P., Bermpeis K., Munhoz D., Sakai K., Seki R., Ohashi H., Esposito G. (2023). Continuous vs Bolus Thermodilution to Assess Microvascular Resistance Reserve. JACC Cardiovasc. Interv..

[71] Gallinoro E., Bertolone D.T., Fernandez-Peregrina E., Paolisso P., Bermpeis K., Esposito G., Gomez-Lopez A., Candreva A., Mileva N., Belmonte M. (2023). Reproducibility of bolus versus continuous thermodilution for assessment of coronary microvascular function in patients with ANOCA. EuroIntervention.

[72] Gutiérrez-Barrios A., Izaga-Torralba E., Rivero Crespo F., Gheorghe L., Cañadas-Pruaño D., Gómez-Lara J., Silva E., Noval-Morillas I., Zayas Rueda R., Calle-Pérez G. (2021). Continuous Thermodilution Method to Assess Coronary Flow Reserve. Am. J. Cardiol..

[73] van’t Veer M., Geven M.C., Rutten M.C., van der Horst A., Aarnoudse W.H., Pijls N.H., van de Vosse F.N. (2009). Continuous infusion thermodilution for assessment of coronary flow: Theoretical background and in vitro validation. Med. Eng. Phys..

[74] Gallinoro E., Candreva A., Colaiori I., Kodeboina M., Fournier S., Nelis O., Di Gioia G., Sonck J., van’t Veer M., Pijls N.H.J. (2021). Thermodilution-derived volumetric resting coronary blood flow measurement in humans. EuroIntervention.

[75] Jeremias A., Kirtane A.J., Stone G.W. (2017). A Test in Context: Fractional Flow Reserve: Accuracy, Prognostic Implications, and Limitations. J. Am. Coll. Cardiol..

[76] De Bruyne B., Pijls N.H., Kalesan B., Barbato E., Tonino P.A., Piroth Z., Jagic N., Möbius-Winkler S., Rioufol G., Witt N. (2012). Fractional flow reserve-guided PCI versus medical therapy in stable coronary disease. N. Engl. J. Med..

[77] Fournier S., Keulards D.C.J., van’t Veer M., Colaiori I., Di Gioia G., Zimmermann F.M., Mizukami T., Nagumo S., Kodeboina M., El Farissi M. (2021). Normal values of thermodilution-derived absolute coronary blood flow and microvascular resistance in humans. EuroIntervention.

[78] Wang B., Gao Y., Zhao Y., Xu C., Zhao S., Li H., Zhang Y., Xu Y. (2023). The spectrum of angiography-derived IMR according to morphological and physiological coronary stenosis in patients with suspected myocardial ischemia. Clin. Cardiol..

[79] Lee J.M., Jung J.H., Hwang D., Park J., Fan Y., Na S.H., Doh J.H., Nam C.W., Shin E.S., Koo B.K. (2016). Coronary Flow Reserve and Microcirculatory Resistance in Patients with Intermediate Coronary Stenosis. J. Am. Coll. Cardiol..

[80] Smilowitz N.R., Toleva O., Chieffo A., Perera D., Berry C. (2023). Coronary Microvascular Disease in Contemporary Clinical Practice. Circ. Cardiovasc. Interv..

[81] Januszek R., Kołtowski Ł., Tomaniak M., Wańha W., Wojakowski W., Grygier M., Siłka W., Jan Horszczaruk G., Czarniak B., Kręcki R. (2024). Implementation of Microcirculation Examination in Clinical Practice-Insights from the Nationwide POL-MKW Registry. Medicina.

[82] Fawaz S., Khan S., Simpson R., Clesham G., Cook C.M., Davies J.R., Karamasis G.V., Keeble T.R. (2023). Invasive Detection of Coronary Microvascular Dysfunction: How It Began, and Where We Are Now. Interv. Cardiol..

[83] Zdzierak B., Zasada W., Dziewierz A., Pawlik A., Rzeszutko Ł., Bartuś S. (2025). Pullback pressure gradient: A novel dimension in coronary physiology. Kardiol. Pol..

[84] Al-Gully J., Oliveri F., Forouzanfar J.P., Montero-Cabezas J.M., Jukema J.W., den Haan M.C., Al Amri I., Bingen B.O. (2025). Prognostic role of con-/discordant coronary flow reserve and microvascular resistance in coronary microvascular disease: A systematic review and network meta-analysis. Open Heart.

[85] Dimitriadis K., Pyrpyris N., Sakalidis A., Beneki E., Chrysohoou C., Aznaouridis K., Tsioufis K. (2025). The prognostic role of microvascular resistance reserve: A systematic review and meta-analysis. Cardiovasc. Revasc. Med..

[86] Zdzierak B., Zasada W., Rakowski T., Krawczyk-Ożóg A., Bartuś S., Surdacki A., Dziewierz A. (2023). Influence of diabetes mellitus on the invasive assessment of myocardial ischemia in patients with coronary artery disease. Pol. Arch. Intern. Med..

[87] Zasada W., Zdzierak B., Rakowski T., Bobrowska B., Krawczyk-Ożóg A., Surowiec S., Bartuś S., Surdacki A., Dziewierz A. (2023). The Impact of Age on the Physiological Assessment of Borderline Coronary Stenoses. Medicina.

[88] Vrints C., Andreotti F., Koskinas K.C., Rossello X., Adamo M., Ainslie J., Banning A.P., Budaj A., Buechel R.R., Chiariello G.A. (2024). 2024 ESC Guidelines for the management of chronic coronary syndromes. Eur. Heart J..

[89] Simova I. (2015). Coronary Flow Velocity Reserve Assessment with Transthoracic Doppler Echocardiography. Eur. Cardiol..

[90] Dimitrow P.P. (2003). Transthoracic Doppler echocardiography–noninvasive diagnostic window for coronary flow reserve assessment. Cardiovasc. Ultrasound.

[91] Tesic M., Beleslin B., Giga V., Jovanovic I., Marinkovic J., Trifunovic D., Petrovic O., Dobric M., Aleksandric S., Juricic S. (2021). Prognostic Value of Transthoracic Doppler Echocardiography Coronary Flow Velocity Reserve in Patients with Asymmetric Hypertrophic Cardiomyopathy. J. Am. Heart Assoc..

[92] Meimoun P., Benali T., Sayah S., Luycx-Bore A., Boulanger J., Zemir H., Tribouilloy C. (2005). Evaluation of left anterior descending coronary artery stenosis of intermediate severity using transthoracic coronary flow reserve and dobutamine stress echocardiography. J. Am. Soc. Echocardiogr..

[93] Carbone A., D’Andrea A., Sperlongano S., Tagliamonte E., Mandoli G.E., Santoro C., Evola V., Bandera F., Morrone D., Malagoli A. (2021). Echocardiographic assessment of coronary microvascular dysfunction: Basic concepts, technical aspects, and clinical settings. Echocardiography.

[94] Bailly M., Thibault F., Metrard G., Courtehoux M., Angoulvant D., Ribeiro M.J. (2023). Precision of Myocardial Blood Flow and Flow Reserve Measurement During CZT SPECT Perfusion Imaging Processing: Intra- and Interobserver Variability. J. Nucl. Med..

[95] D’Antonio A., Assante R., Zampella E., Cantoni V., Green R., Gaudieri V., Mannarino T., Falzarano M., Volpicelli F., Cutillo P. (2025). Prognostic value of myocardial flow reserve by PET imaging in patients with suspected coronary artery disease: A systematic review and meta-analysis. Int. J. Cardiol. Heart Vasc..

[96] Pan C., Yin R., Tang X., Wang T., Hu C. (2023). Prognostic Significance of Myocardial Blood Flow Quantification for Major Adverse Cardiac Events: A Systematic Review and Meta-analysis. Cardiol. Rev..

[97] Kramer C.M., Chandrashekhar Y. (2018). Quantitative Myocardial Perfusion CMR: Is the Game Worth the Candle?. JACC Cardiovasc. Imaging.

[98] Catania R., Quinn S., Rahsepar A.A., Trabzonlu T.A., Bisen J.B., Chow K., Lee D.C., Avery R., Kellman P., Allen B.D. (2025). Quantitative Stress First-Pass Perfusion Cardiac MRI: State of the Art. RadioGraphics.

[99] Pons-Lladó G., Kellman P. (2022). State-of-the-Art of Myocardial Perfusion by CMR: A Practical View. Rev. Cardiovasc. Med..

[100] Borodzicz-Jazdzyk S., Vink C.E.M., Demirkiran A., Hoek R., de Mooij G.W., Hofman M.B.M., Wilgenhof A., Appelman Y., Benovoy M. (2024). Clinical implementation of a fully automated quantitative perfusion cardiovascular magnetic resonance imaging workflow with a simplified dual-bolus contrast administration scheme. Sci. Rep..

[101] Williams M.C., Newby D.E. (2016). CT myocardial perfusion imaging: Current status and future directions. Clin. Radiol..

[102] Rossi A., Wragg A., Klotz E., Pirro F., Moon J.C., Nieman K., Pugliese F. (2017). Dynamic Computed Tomography Myocardial Perfusion Imaging: Comparison of Clinical Analysis Methods for the Detection of Vessel-Specific Ischemia. Circ. Cardiovasc. Imaging.

[103] Nieman K., Balla S. (2020). Dynamic CT myocardial perfusion imaging. J. Cardiovasc. Comput Tomogr..

[104] Otaki Y., Manabe O., Miller R.J.H., Manrique A., Nganoa C., Roth N., Berman D.S., Germano G., Slomka P.J., Agostini D. (2021). Quantification of myocardial blood flow by CZT-SPECT with motion correction and comparison with 15O-water PET. J. Nucl. Cardiol..

[105] Mallet F., Poitrasson-Rivière A., Mariano-Goulart D., Agostini D., Manrique A. (2023). Measuring myocardial blood flow using dynamic myocardial perfusion SPECT: Artifacts and pitfalls. J. Nucl. Cardiol..

[106] Slomka P.J., Patton J.A., Berman D.S., Germano G. (2009). Advances in technical aspects of myocardial perfusion SPECT imaging. J. Nucl. Cardiol..

[107] Gould K.L., Kitkungvan D., Johnson N.P., Nguyen T., Kirkeeide R., Bui L., Patel M.B., Roby A.E., Madjid M., Zhu H. (2021). Mortality Prediction by Quantitative PET Perfusion Expressed as Coronary Flow Capacity with and Without Revascularization. JACC Cardiovasc. Imaging.

[108] Johnson N.P., Kirkeeide R.L., Gould K.L. (2012). Is discordance of coronary flow reserve and fractional flow reserve due to methodology or clinically relevant coronary pathophysiology?. JACC Cardiovasc. Imaging.

[109] Maron D.J., Hochman J.S., Reynolds H.R., Bangalore S., O’Brien S.M., Boden W.E., Chaitman B.R., Senior R., López-Sendón J., Alexander K.P. (2020). Initial Invasive or Conservative Strategy for Stable Coronary Disease. N. Engl. J. Med..

[110] Choi K.H., Lee J.M., Koo B.K., Nam C.W., Shin E.S., Doh J.H., Rhee T.M., Hwang D., Park J., Zhang J. (2018). Prognostic Implication of Functional Incomplete Revascularization and Residual Functional SYNTAX Score in Patients with Coronary Artery Disease. JACC Cardiovasc. Interv..

[111] Jespersen L., Hvelplund A., Abildstrøm S.Z., Pedersen F., Galatius S., Madsen J.K., Jørgensen E., Kelbæk H., Prescott E. (2012). Stable angina pectoris with no obstructive coronary artery disease is associated with increased risks of major adverse cardiovascular events. Eur. Heart J..

[112] Bairey Merz C.N., Pepine C.J., Walsh M.N., Fleg J.L. (2017). Ischemia and No Obstructive Coronary Artery Disease (INOCA): Developing Evidence-Based Therapies and Research Agenda for the Next Decade. Circulation.

[113] Taqueti V.R., Solomon S.D., Shah A.M., Desai A.S., Groarke J.D., Osborne M.T., Hainer J., Bibbo C.F., Dorbala S., Blankstein R. (2018). Coronary microvascular dysfunction and future risk of heart failure with preserved ejection fraction. Eur. Heart J..

[114] Heggie R., Briggs A., Stanley B., Good R., Rocchiccioli P., McEntegart M., Watkins S., Eteiba H., Shaukat A., Lindsay M. (2021). Stratified medicine using invasive coronary function testing in angina: A cost-effectiveness analysis of the British Heart Foundation CorMicA trial. Int. J. Cardiol..

[115] Waheed N., Elias-Smale S., Malas W., Maas A.H., Sedlak T.L., Tremmel J., Mehta P.K. (2020). Sex differences in non-obstructive coronary artery disease. Cardiovasc. Res..

[116] Mehta P.K., Quesada O., Al-Badri A., Fleg J.L., Volgman A.S., Pepine C.J., Merz C.N.B., Shaw L.J. (2022). Ischemia and no obstructive coronary arteries in patients with stable ischemic heart disease. Int. J. Cardiol..

[117] Sinha A., Rahman H., Perera D. (2020). Coronary microvascular disease: Current concepts of pathophysiology, diagnosis and management. Cardiovasc. Endocrinol. Metab..

[118] Boerhout C.K.M., Namba H.F., Liu T., Beijk M.A.M., Damman P., Meuwissen M., Ong P., Sechtem U., Appelman Y., Berry C. (2025). Rationale and design of the ILIAS ANOCA clinical trial: A blinded-arm controlled trial for routine ad-hoc coronary function testing. Am. Heart J..

[119] Szolc P., Guzik B., Niewiara Ł., Kleczyński P., Bernacik A., Diachyshyn M., Stąpór M., Żmudka K., Legutko J. (2025). Tailored treatment of specific diagnosis improves symptoms and quality of life in patients with myocardial Ischemia and Non-obstructive Coronary Arteries. Sci. Rep..

[120] Neglia D., Michelassi C., Trivieri M.G., Sambuceti G., Giorgetti A., Pratali L., Gallopin M., Salvadori P., Sorace O., Carpeggiani C. (2002). Prognostic role of myocardial blood flow impairment in idiopathic left ventricular dysfunction. Circulation.

[121] Dryer K., Gajjar M., Narang N., Lee M., Paul J., Shah A.P., Nathan S., Butler J., Davidson C.J., Fearon W.F. (2018). Coronary microvascular dysfunction in patients with heart failure with preserved ejection fraction. Am. J. Physiol. Heart Circ. Physiol..

[122] Sharka I., Panichella G., Grigoratos C., Muca M., De Gori C., Keilberg P., Novani G., Barra V., Hlavata H., Bianchi M. (2025). Myocardial Perfusion Imaging with Cardiovascular Magnetic Resonance in Nonischemic Cardiomyopathies: An In-Depth Review of Techniques and Clinical Applications. Medicina.

[123] Toya T., Corban M.T., Park J.Y., Ahmad A., Özcan I., Sebaali F., Sara J.D.S., Gulati R., Lerman L.O., Lerman A. (2021). Prognostic impact and clinical outcomes of coronary flow reserve and hyperaemic microvascular resistance. EuroIntervention.

[124] Sadeghi R., Adnani N., Sohrabi M.R., Alipour Parsa S. (2013). Risk of sudden cardiac death. ARYA Atheroscler..

[125] Niewiara Ł., Kleczyński P., Guzik B., Szolc P., Baran J., Podolec J., Diachyshyn M., Żmudka K., Legutko J. (2024). Impaired coronary flow reserve in patients with poor type 2 diabetes control: Preliminary results from prospective microvascular dysfunction registry. Cardiol. J..

[126] Kawata T., Daimon M., Hasegawa R., Toyoda T., Sekine T., Himi T., Uchida D., Miyazaki S., Hirose K., Ichikawa R. (2013). Prognostic value of coronary flow reserve assessed by transthoracic Doppler echocardiography on long-term outcome in asymptomatic patients with type 2 diabetes without overt coronary artery disease. Cardiovasc. Diabetol..

[127] Dahl J.N., Nielsen M.B., Birn H., Rasmussen L.D., Ivarsen P., Svensson M., Bangalore S., Bøttcher M., Winther S. (2022). Prognostic value of computed tomography derived fractional flow reserve for predicting cardiac events and mortality in kidney transplant candidates. J. Cardiovasc. Comput Tomogr..

[128] Itakura R., Kuramitsu S., Kikuchi J., Kawase Y., Mizukami T., Shinozaki T., Horie K., Takashima H., Terai H., Kikuta Y. (2023). Prognostic Impact of Renal Function on 5-Year Outcomes After Fractional Flow Reserve-Guided Deferral of Revascularization. J. Am. Heart Assoc..

[129] Li Z., Zhu S., Tang W., Zhang H., Le W., Luo S., Zhou C., Wang Y., Xu S., Hu W. (2025). Clinical features and two-year outcomes in systemic lupus erythematosus patients with heart failure and reduced, mid-range and preserved ejection fractions. Lupus.

[130] Liao K.P., Huang J., He Z., Cremone G., Lam E., Hainer J.M., Morgan V., Bibbo C., Di Carli M. (2021). Coronary Microvascular Dysfunction in Rheumatoid Arthritis Compared to Diabetes Mellitus and Association with All-Cause Mortality. Arthritis Care Res..

[131] Ikonomidis I., Iliodromitis E.K., Tzortzis S., Antoniadis A., Paraskevaidis I., Andreadou I., Fountoulaki K., Farmakis D., Kremastinos D.T., Anastasiou-Nana M. (2010). Staccato reperfusion improves myocardial microcirculatory function and long-term left ventricular remodelling: A randomised contrast echocardiography study. Heart.

[132] Paraskevaidis I.A., Iliodromitis E.K., Ikonomidis I., Rallidis L., Hamodraka E., Parissis J., Andoniadis A., Tzortzis S., Anastasiou-Nana M. (2012). The effect of acute administration of statins on coronary microcirculation during the pre-revascularization period in patients with myocardial infraction. Atherosclerosis.

[133] Tzortzis S., Ikonomidis I., Triantafyllidi H., Trivilou P., Pavlidis G., Katsanos S., Katogiannis K., Birba D., Thymis J., Makavos G. (2020). Optimal Blood Pressure Control Improves Left Ventricular Torsional Deformation and Vascular Function in Newly Diagnosed Hypertensives: A 3-Year Follow-up Study. J. Cardiovasc. Transl. Res..

[134] Ling H., Fu S., Xu M., Wang B., Li B., Li Y., Liu X., Zhang X., Wang Q., Li A. (2024). Ranolazine for improving coronary microvascular function in patients with nonobstructive coronary artery disease: A systematic review and meta-analysis with a trial sequential analysis of randomized controlled trials. Quant. Imaging Med. Surg..

[135] Yong J., Tian J., Yang X., Xing H., He Y., Song X. (2020). Effects of Oral Drugs on Coronary Microvascular Function in Patients Without Significant Stenosis of Epicardial Coronary Arteries: A Systematic Review and Meta-Analysis of Coronary Flow Reserve. Front. Cardiovasc. Med..

[136] Vervaat F.E., de Vos A., Schenk J., Tonino P.A.L., Wijnbergen I.F. (2025). Treatment Modalities for Angina with Non-Obstructive Coronary Arteries (ANOCA): A Systematic Review and Meta-Analysis. J. Clin. Med..

[137] Ikonomidis I., Papadavid E., Makavos G., Andreadou I., Varoudi M., Gravanis K., Theodoropoulos K., Pavlidis G., Triantafyllidi H., Moutsatsou P. (2017). Lowering Interleukin-12 Activity Improves Myocardial and Vascular Function Compared with Tumor Necrosis Factor-a Antagonism or Cyclosporine in Psoriasis. Circ. Cardiovasc. Imaging.

[138] Ikonomidis I., Lekakis J.P., Nikolaou M., Paraskevaidis I., Andreadou I., Kaplanoglou T., Katsimbri P., Skarantavos G., Soucacos P.N., Kremastinos D.T. (2008). Inhibition of interleukin-1 by anakinra improves vascular and left ventricular function in patients with rheumatoid arthritis. Circulation.

[139] Matteucci A., Mariani M.V., Sgarra L., Bonanni M., Frazzetto M., La Fazia V.M., Pierucci N., Lavalle C., Pandozi C., Nardi F. (2024). Epicardial Ablation for Arrhythmogenic Disorders in Patients with Brugada Syndrome. Biomedicines.

[140] Matteucci A., Massaro G., Mamas M.A., Biondi-Zoccai G. (2021). Expanding the role of fractional flow reserve derived from computed tomography (FFRCT) for the non-invasive imaging of patients with coronary stents: Rise of the machines?. Eur. Radiol..

[141] Cipollone P., Pierucci N., Matteucci A., Palombi M., Laviola D., Bruti R., Vinciullo S., Bernardi M., Spadafora L., Cersosimo A. (2025). Artificial Intelligence in Cardiac Electrophysiology: A Comprehensive Review. J. Pers. Med..

[142] Andersen B.K., Sejr-Hansen M., Maillard L., Campo G., Råmunddal T., Stähli B.E., Guiducci V., Serafino L.D., Escaned J., Santos I.A. (2024). Quantitative flow ratio versus fractional flow reserve for coronary revascularisation guidance (FAVOR III Europe): A multicentre, randomised, non-inferiority trial. Lancet.

